# PCR/RFLP-Based Analysis of Genetically Distinct *Plasmodium vivax* Population of *Pvmsp-3α* and *Pvmsp-3β* genes in Pakistan

**DOI:** 10.1186/1475-2875-13-355

**Published:** 2014-09-09

**Authors:** Shahid Niaz Khan, Asif Khan, Sanaullah Khan, Sultan Ayaz, Sobia Attaullah, Jabbar Khan, Muhammad Asim Khan, Ijaz Ali, Abdul Haleem Shah

**Affiliations:** Department of Zoology, Kohat University of Science and Technology, Kohat, 26000 Khyber Pakhtunkhwa Pakistan; Department of Zoology, Islamia College (A Public Sector University), Peshawar, Khyber Pakhtunkhwa Pakistan; Department of Biological Sciences, Gomal University D I Khan, Kragujevac, Khyber-Pakhtunkhwa Pakistan; Institute of Biotechnology and Genetic Engineering, Agriculture University Peshawar, Kragujevac, Khyber Pakhtunkhwa Pakistan

**Keywords:** *Plasmodium vivax*, PCR/RFLP, Genetic polymorphism, MSP3*α* and MSP3*β* genes

## Abstract

**Background:**

*Plasmodium vivax* is one of the widespread human malarial parasites accounting for 75% of malaria epidemics. However, there is no baseline information about the status and nature of genetic variation of *Plasmodium* species circulating in various parts of Pakistan. The present study was aimed at observing the molecular epidemiology and genetic variation of *Plasmodium vivax by* analysing its merozoite surface protein-3α (*msp-3α*) and merozoite surface protein-3β (*msp-3β*) genes, by using suballele, species-specific, combined nested PCR/RFLP detection techniques.

**Methods:**

A total of 230 blood samples from suspected subjects tested slide positive for vivax malaria were collected from Punjab, Sindh, Khyber Pakhtunkhwa, and Balochistan during the period May 2012 to December 2013. Combined nested PCR/RFLP technique was conducted using *Pvmsp-3α* and *Pvmsp-3β* genetic markers to detect extent of genetic variation in clinical isolates of *P. vivax* in the studied areas of Pakistan.

**Results:**

By PCR, *P. vivax,* 202/230 (87.82%), was found to be widely distributed in the studied areas. PCR/RFLP analysis showed a high range of allelic variations for both *msp-3α* and *msp-3β* genetic markers of *P. vivax,* i.e., 21 alleles for *msp-3α* and 19 for *msp-3β*. Statistically a significant difference (p ≤ 0.05) was observed in the genetic diversity of the suballelic variants of *msp-3α* and *msp-3β* genes of *P. vivax.*

**Conclusion:**

It is concluded that *P. vivax* populations are highly polymorphic and diverse allelic variants of *Pvmsp-3α* and *Pvmsp-3β* are present in Pakistan.

## Background

Malaria is the major threat to public health and economic development in many nations [1]. It kills more than a million people a year, and approximately 40% of the world’s populations live in malarious countries [2]. *Plasmodium vivax* is the most widespread species of human malaria parasites in the world and is endemic in many countries of Asia, Central and South America, the Middle East, and parts of Africa, with an estimated burden of 70-80 million cases annually [3, 4]. In Pakistan *P. vivax* is the common malarial species, contributing to 70% of the malaria burden [5]. Although *P. vivax* is highly prevalent, it has received scant scientific attention in Pakistan, thus, a paucity of baseline data exists on various aspects of *P. vivax*, such as population structure and drug resistance patterns.

Genetic diversity is defined as the total number of heritable characteristics in the genetic make-up of the species [6]. Studies have shown that *Plasmodium* species show diversity in several parameters, including morphology, biochemistry, relapse patterns, symptoms, course and duration of infection, immunological responses, drug resistance, and transmissibility by anopheline vectors. However, the extent of genetic diversity observed depends on the transmission rates, immune pressure and natural selection of the parasite in an area [7]. Usually in endemic areas, where transmission rates are high, extensively diverse variants are in circulation, posing a threat to public health, since such variants have possibly acquired increased virulence and resistance to drugs in order to fit and survive [8].

Studies of the population structure of malaria parasites are important for understanding the evolution of parasite virulence and the role of parasite diversity in malaria transmission, and for designing control tools, including vaccines, as well as evaluating the impact of malaria control measures [9, 10].

*Pvmsp-3α* and *Pvmsp-3β* have been used as markers in population genetic studies worldwide [11–14]. The best approach for detecting genetic diversity is to analyse more than one marker gene, because by doing so the probability of different clones sharing the same genotype accidentally, is substantially reduced [15]. Accordingly, it is suggested that analysis of both *Pvmsp-3α* and *Pvmsp3β* genes of *P. vivax* enables greater ability in identifying parasite haplotypes and detecting mixed strain infections [11, 12]. Limited data regarding the genetic diversity of *Pvmsp-3α* and *Pvmsp-3β* is available from one endemic area of Khyber Pakhtunkhwa province of Pakistan [16], it is therefore, hypothesized that new allelic variants may be present in other provinces of the country. This study will seek to elaborate on the genetic polymorphism of *P. vivax* malaria isolates and compare them with those reported from other parts of the world.

## Methods

### Sample collection

This study was conducted with the approval of the Medical Superintendents of source hospitals and the ethics committee of Kohat University of Science and Technology (KUST) Kohat Pakistan. A total of 230 blood samples were collected, after the informed consent from symptomatic patients, with the assessment of malaria control laboratories in the district headquarters hospitals of the four provinces: Punjab, Sindh, Khyber Pakhtunkhwa, and Balochistan during May 2012 to December 2013. Out of these 230 samples, 58 were collected from Punjab, 54 from Sindh, 68 from Khyber Pakhtunkhwa, and 50 from Balochistan. All the samples were screened for the detection of malarial parasites by microscope and PCR.

### DNA purification

DNA of blood samples were extracted with the help of DNA extraction kit (GF1, Vivantus, USA).

### PCR amplification for *Plasmodium vivax*identification

The nested PCR amplification procedures used by Snounou *et al.* and Singh *et al.* [17, 18] were applied for amplification and detection of the 18S rRNA type A gene size 1.2 Kb, using species-specific primers rVIV1 5′-CGCTTCTAGCTTAATCCACATAACTGATAC-3′ and rVIV2 5′-ACTTCCAAGCCGAAGCAAAGAAAGTCCTTA-3′ for the detection of *P. vivax*.

### PCR of *Pvmsp-3α*genes

By using PCR technique, the allelic variants of *P. vivax* merozoite surface protein-3α were detected by standard protocol of Bruce *et al.* [11]. Primary PCR of *Pvmsp-3α* was performed in 20 ml using 3 ml of DNA, using primers P1 (5′-CAGCAGACACCATTTAAGG-3′) and P2 (5′-CCGTTTGTTGATTAGTTGC-3′), while nested reactions were done in 20 μl with primers N1 (5′-GACCAGTGTGATACCATTAACC-3′) and N2 (5′-ATACTGGTTCTTCGTCTTCA GG-3′) using 3 μl of the primary PCR product. *Taq* DNA polymerase (2.6 units) was used and the following cyclic conditions were performed: 95°C for 3 min, 94°C for 30 sec, 58°C for 30 sec, 68°C for 2.5 min (2, 30 times) and 72°C for 5 min. Two per cent agarose gel was stained with ethidium bromide in order to visualize the PCR separated product, under UV illumination. Gel documentation system (Clearver Scientific, USA) was used for the photography.

### RFLP analysis of *Pvmsp-3α*genes PCR products

For the RFLP analysis of *Pvmsp-3α* genes, *Alu1* restriction enzyme (Fermentas, USA) was used. The total volume of the reaction medium was kept 20 μl having the following reagents *Alu1* buffer (1.2 μl), PCR water (9.8 μl), *Alu1* enzyme (1.0 μl) and product of *msp-*3α gene (8.0 μl).

### PCR of *Pvmsp-3β*genes

The allelic variants of *P. vivax* merozoite’s surface protein-3 were detected by PCR analysis, using standard protocol of Yang *et al*. [12]. For *Pvmsp-3β,* primary PCR was performed in 20 μl using 3 μl of DNA using primers P1 (5′-GTATTCTTCGCAACACTC-3′) and P2 (5′-CTTCTGATGTTATTTCCAG-3′). Nested reactions were done in 20 ml with primers N1 (5′-CGAGGGGCGAAATTGTAAACC-3′) and N2 (5′-GCTGCTTCTTTTGCAAAGG-3′) using 1 μl of the primary PCR product as the template. *Taq* DNA polymerase (2.6 units) (Vivantis, USA) was used and the following cyclic conditions were performed: 94°C for 20 sec, 54°C for 30 sec, 68°C for 2.5 min, 35 cycles. Agarose was used to visualize the PCR product under UV illumination. Gel documentation system (Clearver Scientific, USA) was used for the photography.

### RFLP analysis of *Pvmsp-3β*PCR products

The RFLP analysis of *Pvmsp-*3β gene was carried out, using *Pest* 1 restriction enzyme (Fermentas, USA) described by Yang *et al.* [12]. The total volume of the reaction medium was kept as 20 μl. The master mix for RFLP analysis was composed of *Pest* 1 Buffer 1.2 μl, PCR water 9.8 μl, *Pest* 1 enzyme 1.0 μl and product of *msp-3β* gene 8.0 μl.

### Statistical analysis

The data were statistically analysed by using SPSS (Version 20) with application of Kruskal Wallis test. The p value less than 0.05 was assumed to be statistically significant.

## Results

**Figure 1:**
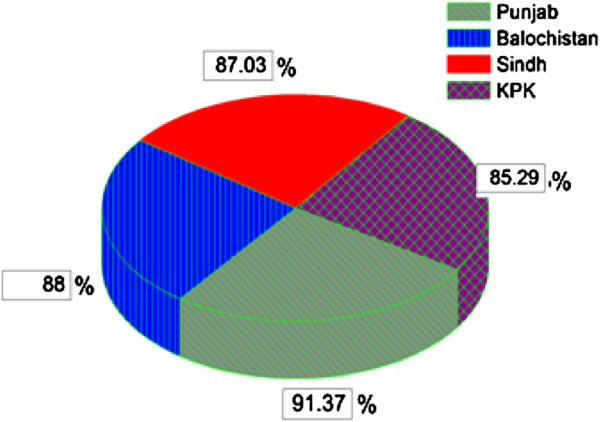
**Overall prevalence of vivax malaria in Pakistan by PCR.**

Out of the total 230 samples, 202 samples were found positive for *P. vivax* both by microscopy and PCR. The distribution of the positive samples in the four provinces of Pakistan remained: 58/68 (85.29%) for Khyber Pakhtunkhwa, 53/58 (91.37%) for Punjab, 47/54 (87.03%) for Sindh and 44/50 (88%) for Balochistan (Figure 1).

### Analysis of *pvmsp-*3α gene by PCR /RFLP

**Figure 2:**
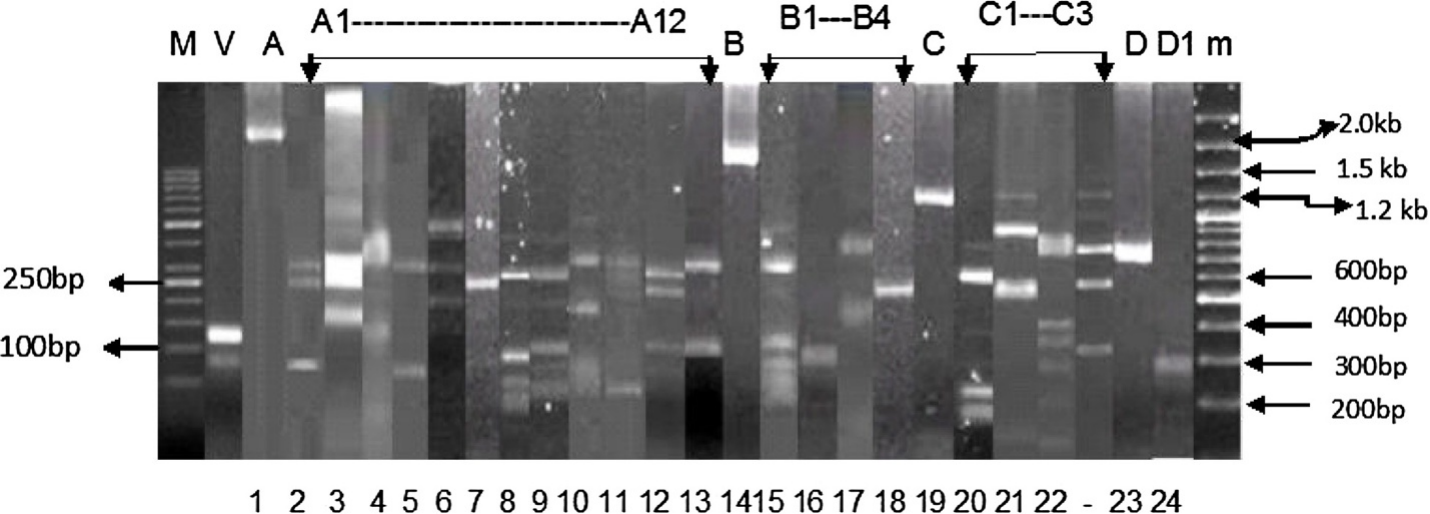
**PCR/RFLP analysis of** ***P. vivax msp-3α*** **gene.** V shows *P. vivax* (the amplified product is 120 bp). **A**, **B**, **C** and **D** indicate the main allele variants, while **A**, **B**, **C** and **D** with numbers indicate subtypes of the main allele variants. M is a 50 base pairs DNA marker and m is a 1 kilo base pairs DNA marker.

A sum of 130/202 samples of blood were successfully amplified for *pvmsp*-3α gene and were identified for different genotypes of the aforementioned gene. Three major allelic variants of different sizes, i.e., 2.2 kb (Type-A), 2.0 kb (Type-B), 1.1 kb (Type-C) were detected. Besides these, an unusual band size of about 0.75 kb, named as Type-D and mixed genotypes designated as M were also observed (Figure 2). Out of the 130 amplified samples, the frequency of the different types of alleles remained 64/130 (49.29%) for Type-A, 27/130 (20.76%) for Type-B, 27/130 (20.76%) for Type-C, 9/130 (6.92 %) for Type-D, and 3/130 (2.30 %) for mixed. Overall 12 suballelic variants (A1-A12) for Type A, 5 (B1-B5) for Type B and 4 (C1-C4) for Type-C were observed. The alleles A3, A9, B1, B2, and C2 were frequently observed in all provinces of Pakistan (Table 1).

**Table 1:** **Size, RFLP pattern and allelic frequency of** ***Pvmsp*** **-** ***3α*** **gene**

Size variation	RFLP by ***Alu***1	Alleles	Data analysis n (%) frequency distribution of sub-allelic variants				
			Punjab	Sindh	Khyber Pakhtunkhwa	Balochistan	Total
			(n = 32)	(n = 27)	(n = 41)	(n = 30)	(n = 130)
A	A1—A12	1	2 (6.25)	2 (7.40)	2 (4.87)	2 (6.66)	8 (6.15)
		2	2 (6.25)	1 (3.70)	2 (4.87)	1 (3.33)	6 (4.61)
		3	3 (9.37)	2 (7.40)	4 (9.75)	2 (6.66)	11 (8.46)
		4	2 (6.25)	1 (3.70)	2 (4.87)	1 (3.33)	6 (4.61)
		5	1 (3.12)	1 (3.70)	1 (2.43)	1 (3.33)	4 (3.07)
		6	2 (6.25)	1 (3.70)	1 (2.43)	1 (3.33)	4 (3.07)
		7	0 (0.00)	1 (3.70)	1 (2.43)	1 (3.33)	3 (2.30)
		8	1 (3.12)	0 (0.00)	1 (2.43)	1 (3.33)	3 (2.30)
		9	3 (9.37)	3 (11.11)	4 (9.75)	3 (10)	13 (10)
		10	0 (0.00)	0 (0.00)	1 (2.43)	1 (3.33)	2 (1.53)
		11	2 (6.25)	0 (0.00)	1 (2.43)	1 (3.33)	3 (2.30)
		12	0 (0.00)	0 (0.00)	1 (2.43)	0 (0.00)	1 (0.76)
B	B1—B5	13	3 (9.37)	3 (11.11)	3 (7.31)	2 (6.66)	11 (8.46)
		14	2 (6.25)	3 (11.11)	3 (7.31)	3 (10)	11(8.46)
		15	1 (3.12)	1 (3.70)	1 (2.43)	1 (3.33)	4 (3.07)
		16	0 (0.00)	0 (0.00)	1 (2.43)	0 (0.00)	1 (0.76)
		17	0 (0.00)	0 (0.00)	0 (0.00)	0 (0.00)	0 (0.00)
C	C1—C4	18	2 (6.25)	1 (3.70)	2 (4.87)	1 (3.33)	6 (4.61)
		19	4 (12.5)	3 (11.11)	4 (9.75)	4 (13.33)	15 (11.53)
		20	2 (6.25)	1 (3.70)	2 (4.87)	1 (3.33)	6 (4.61)
		21	0 (0.00)	0 (0.00)	0 (0.00)	0 (0.00)	0 (0.00)
D	D1	22	2 (6.25)	2 (7.40)	3 (7.31)	2 (6.66)	9 (6.92)
M	M1	23	0 (0.00)	1 (3.70)	1 (2.43)	1 (3.33)	3 (2.30)
Total frequency (%)			32 (24.61)	27 (20.76)	41 (31.53)	30 (23.07)	130 (64.35)
Mean ± SE			1.48 ± 0.80	1.30 ± 0.96	1.17 ± 0.92	1.78 ± 0.79	
Kruskal wallis test			p < 0.05				

n = The number of subtypes of alleles that bear *Alu*1 RFLP pattern.

### Analysis of *pvmsp*-3β gene by PCR/RFLP

**Figure 3:**
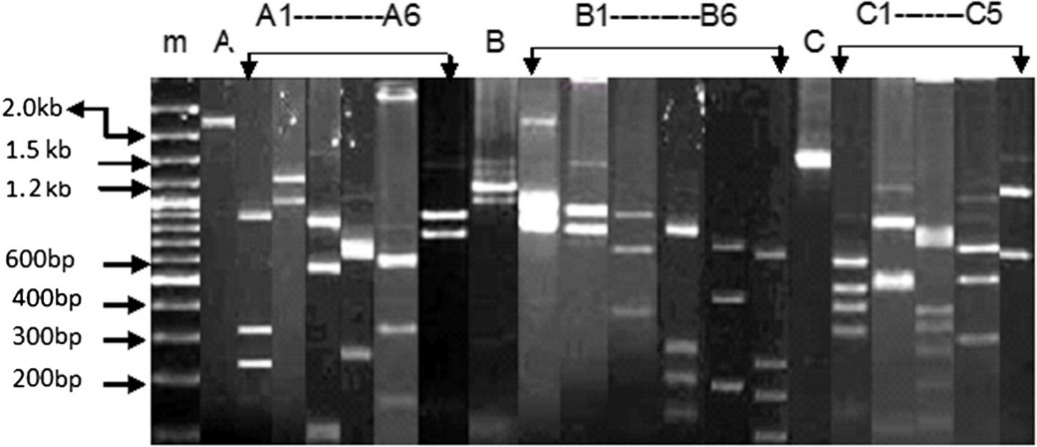
**PCR/RFLP analysis of** ***P. vivax msp-3β*** **gene. A**, **B** and **C** indicate the main allele variants, while **A**, **B** and **C** with numbers indicate subtypes of the main allele variants. m is a 1 kilo base pairs DNA marker.

In the current study 72/202 samples of blood had been successfully amplified for *pvmsp*-3*β* gene with a high frequency of polymorphism among their different allelic variants. The amplified DNA fragments for these alleles were classified according to their size variation. Based on variation in size of the fragments, the amplified samples could be differentiated into three allele sizes: for Type A (2.0-2.5 kb), Type B (1.6-2.0 kb) and Type C (1.5 kb) (Figure 3).

In all these amplified samples, a couple of samples reflect more than one band of different sizes. These multiple bands show mixed infection, which revealed 2.77**%** of the allelic variants. Out of the total 72 amplified samples, 16 (22.22%) isolates for Type-A, 32 (44.44%) for Type-B and 22 (30.55%) for Type-C were observed. Among these isolates Type-B was found to have highest per cent frequency and Type-A was found the lowest in per cent frequency (Table 2).

**Table 2:** **Size, RFLP pattern and allelic frequency of** ***Pvmsp-3β*** **gene**

Size variation	RFLP by***Pst1***	Alleles	Data analysis n (%) frequency distribution of sub-allelic variants				
			Punjab	Sindh	Khyber Pakhtunkhwa	Balochistan	Total
			(n = 21)	(n = 20)	(n = 17)	(n = 14)	(n = 72)
A	A1—A7	A1	1(4.76)	1 (5)	0 (0.00)	1 (7.14)	3 (4.16)
		A2	1(4.76)	1 (5)	0 (0.00)	0 (0.00)	2 (2.77)
		A3	1(4.76)	1 (5)	1 (5.88)	0 (0.00)	3 (4.16)
		A4	1(4.76)	2 (10)	1 (5.88)	1 (7.14)	5 (6.94)
		A5	1(4.76)	0 (0.00)	1 (5.88)	0 (0.00)	2 (2.77)
		A6	0 (0.00)	0 (0.00)	1 (5.88)	0 (0.00)	1 (1.38)
		A7	-	-	-	-	-
B	B1—B10	B1	1(4.76)	1 (5)	1 (5.88)	1 (7.14)	4 (5.55)
		B2	2 (9.52)	2 (10)	2 (11.76)	2 (14.28)	8 (11.11)
		B3	2 (9.52)	2 (10)	0 (0.00)	1 (7.14)	5 (6.94)
		B4	1(4.76)	1 (5)	1 (5.88)	0 (0.00)	3 (4.16)
		B5	1(4.76)	1 (5)	1 (5.88)	1 (7.14)	4 (5.55)
		B6	2 (9.52)	3 (15)	2 (11.76)	0 (0.00)	7 (9.72)
		B7	0 (0.00)	0 (0.00)	0 (0.00)	1 (7.14)	1 (1.38)
		B8	-	-	-	-	-
		B9	-	-	-	-	-
		B10	-	-	-	-	-
C	C1—C4	C1	2 (9.52)	2 (10)	2 (11.76)	1 (7.14)	7 (9.72)
		C2	2 (9.52)	1 (5)	1 (5.88)	1 (7.14)	5 (6.94)
		C3	1(4.76)	1 (5)	0 (0.00)	1 (7.14)	3 (4.16)
		C4	2 (9.52)	1 (5)	1 (5.88)	1 (7.14)	5 (6.94)
		C5	0 (0.00)	0 (0.00)	1 (5.88)	1 (7.14)	2 (2.77)
		C6	-	-	-	-	-
M	M1	M1	0 (0.00)	0 (0.00)	1 (5.88)	1 (7.14)	2 (2.77)
Total frequency (%)			21 (29.16)	20 (27.77)	17 (23.61)	14 (19.44)	72 (35.64)
Mean ± SE			1.11 ± 0.17	1.05 ± 0.19	0.89 ± 0.15	0.74 ± 0.13	
Kruskal wallis test			p < 0.05				

n = The number of subtypes of alleles that bear *Pst*1 RFLP pattern.

In this study *pvmsp*-3*β* gene was analysed using *Pst*1 restriction enzyme and 7 suballelic variants, (A1-A7) for Type-A, 10 (B1-B10) for Type-B and 6 (C1-C6) for Type-C were observed. The suballeles B7-B10 were not found in the present study (Table 2). The different samples resolved through *pst*1 restriction enzyme, show a high polymorphism in size and distribution among samples (Figure 3).

## Discussion

Pakistan is endemic for malaria and there are reports showing the distribution of various allelic variants of *P. vivax*. So, this study was aimed with the main objectives of identifying the new variants and to observe the epidemiology of level of *pvmsp*-*3α* and *pvmsp*-*3β* polymorphism of *P. vivax* in Pakistan. In the current study four distinct sizes of PCR products for *Pvmsp-3α* were detected. Among these, three different major allelic variants of different sizes, i.e., 2.2 kb (Type-A), 2.0 kb (Type-B), 1.1 kb (Type-C) along with unusual band size of almost 0.75 kb or 300 bp name as Type-D and mixed genotypes designated as M, were observed in the study areas, while in Thailand [13], Afghanistan [19] and the Federally Administered Tribal Areas (FATA) of Pakistan [20], only three allele sizes (A, B and C) were obtained for this gene.

The frequency of the different types of alleles remained 64/130 (49.29%) for Type-A, 27/130 (20.76%) for Type-B, 27/130 (20.76%) for Type-C, 9/130 (6.92%) for Type-D, and 3/130 (2.30%) for mixed, comparing with the study of Khatoon *et al.* where 82% (41/50) isolates having allele sizes 1.9 kb (Type-A), 6% (3/50) with the 1.5 kb fragment (Type-B), 8% (4/50) with the 1.2 kb fragment (Type-C), 2% (1/50) with the 0.3 kb fragment (Type-D) and one isolate (2%) exhibited mixed-strain infection [16].

In this study restriction digestion of the *Pvmsp-3α* PCR product with *Alu*I yielded 12 suballelic variants types: (A1-A12) for Type-A, 5 (B1-B5) for Type-B and 4 (C1-C4) for Type-C were observed. The alleles A3 11 (8.46%) and A9 13 (10%) for Type-A, allele B1 and B2 11 (8.46%) for Type-B and C2 15 (11.53%) for Type-C were frequently observed in all provinces of Pakistan, while in the similar study carried out by Khatoon *et al*., 12 different alleles designated as A1-A7, B1, B2, C1, C2, and D, allele A3 being the most abundant 12/50 (24%) [16].

The PCR-RFLP patterns for *P. vivax Pvmsp-3β* gene produced three categories Type-A (2.0-2.5 kb), Type-B (1.6-2.0 kb) and Type-C (1.5 kb). Similarly, in northwest Thailand, three distinct allele types (A, B and C (~0.65 kb)) were found [12]. In contrast, studies carried out in district Bannu by Khatoon *et al*. found two types of allele A (1.7-2.2 kb) and B (1.4-1.5 kb) of size polymorphisms for *P. vivax Pvmsp-3β gene* [16]. Compared with *P. vivax* isolates from previous work conducted in Asia, the results of the current study further confirmed the existence of small geographic differentiation among *P. vivax* populations. An earlier study in western Thailand showed that the B type of *pvmsp*-3β is more abundant (60.4%) than other types, whereas in Chinese Bengbu and Guangxi samples, both A and B types were similarly prevalent [12]. According to Zhong *et al.*, the Type-A allele was the most abundant in all four parasite populations (>57% more abundant than other types), i.e., Anhui, Hainan, Yunnan, and Myanmar [21]. In the current study, Type-B was found to have the highest per cent frequency and Type-A was found to have lowest per cent frequency.

Data from PCR-RFLP of the *Pvmsp-3α* and *Pvmsp-3β* loci showed that 5.02% of the Pakistani *P. vivax* isolates exhibited mixed-strain infections, which is comparable to sites in Bengbu (China) (5.6%) and is lower than observed in Thailand (20.5%) [12] and FATA of Pakistan (30%), which directly shares a border with Afghanistan [20]. In northern Iran [20] and Hongshuihe (China), no mixed genotypes were detected [12]. Apart from differences in transmission intensities in different areas, the observed variations in parasite types could be attributed to factors such as sampling bias, host immune selective pressure on particular types and/or spatiotemporal changes in the availability of different mosquito species that can transmit specific parasite types in a particular area over different times or seasons, with areas that share the same mosquito types tending to have similar parasite types [20].

## Conclusion

This study indicates that *P.vivax* populations in four provinces of Pakistan: Punjab, Sindh, Khyber Pakhtunkhwa, and Balochistan, are highly diverse. Such heterogeneity might cause differences in parasite virulence, transmissibility and responses to chemotherapy, with important implications for malaria control measures in this populous region.
